# Is early detection of abused children possible?: a systematic review of the diagnostic accuracy of the identification of abused children

**DOI:** 10.1186/1471-2431-13-202

**Published:** 2013-12-05

**Authors:** Marion Bailhache, Valériane Leroy, Pascal Pillet, Louis-Rachid Salmi

**Affiliations:** 1 CHU de Bordeaux, Pole de pediatrie, F-33000 Bordeaux, France; 2 Centre INSERM U897-Epidemiologie-Biostatistique, University Bordeaux, ISPED, F-33000 Bordeaux, France; 3 Centre INSERM U897-Epidemiologie-Biostatistique, INSERM, ISPED, F-33000 Bordeaux, France; 4 CHU de Bordeaux, Pole de sante publique, Service d’information medicale, F-33000 Bordeaux, France

**Keywords:** Child abuse, Child neglect, Systematic review, Diagnostic accuracy

## Abstract

**Background:**

Early detection of abused children could help decrease mortality and morbidity related to this major public health problem. Several authors have proposed tools to screen for child maltreatment. The aim of this systematic review was to examine the evidence on accuracy of tools proposed to identify abused children before their death and assess if any were adapted to screening.

**Methods:**

We searched in PUBMED, PsycINFO, SCOPUS, FRANCIS and PASCAL for studies estimating diagnostic accuracy of tools identifying neglect, or physical, psychological or sexual abuse of children, published in English or French from 1961 to April 2012. We extracted selected information about study design, patient populations, assessment methods, and the accuracy parameters. Study quality was assessed using QUADAS criteria.

**Results:**

A total of 2 280 articles were identified. Thirteen studies were selected, of which seven dealt with physical abuse, four with sexual abuse, one with emotional abuse, and one with any abuse and physical neglect. Study quality was low, even when not considering the lack of gold standard for detection of abused children. In 11 studies, instruments identified abused children only when they had clinical symptoms. Sensitivity of tests varied between 0.26 (95% confidence interval [0.17-0.36]) and 0.97 [0.84-1], and specificity between 0.51 [0.39-0.63] and 1 [0.95-1]. The sensitivity was greater than 90% only for three tests: the absence of scalp swelling to identify children victims of inflicted head injury; a decision tool to identify physically-abused children among those hospitalized in a Pediatric Intensive Care Unit; and a parental interview integrating twelve child symptoms to identify sexually-abused children. When the sensitivity was high, the specificity was always smaller than 90%.

**Conclusions:**

In 2012, there is low-quality evidence on the accuracy of instruments for identifying abused children. Identified tools were not adapted to screening because of low sensitivity and late identification of abused children when they have already serious consequences of maltreatment. Development of valid screening instruments is a pre-requisite before considering screening programs.

## Background

The World Health Organization (WHO) defines child maltreatment as “all forms of physical and/or emotional ill-treatment, sexual abuse, neglect or negligent treatment or commercial or other exploitation, resulting in actual or potential harm to the child’s health, survival, development or dignity” [1]. It is a major public health issue worldwide. Gilbert et al. estimated that every year in high-income countries about 4 to 16% of children were physically abused, one in ten was neglected or psychologically abused, and between 5 and 10% of girls and up to 5% of boys were exposed to penetrative sexual abuse during childhood [2]. Child maltreatment can cause death of the child or major consequences on mental and physical health, such as post-traumatic stress disorder and depression, in childhood or adulthood [2]. WHO estimated that 155 000 deaths in children younger than 15 years occurred worldwide in 2000 as a result of abuse or neglect [3].

In France, a retrospective study carried out in three regions from 1996 to 2000 showed that many children who died from abuse were not identified as abused before their deaths. After excluding clear neonaticides, 25 of 53 (47%) infants who died from suspicious or violent death had signs of prior abuse, such as fractures of different ages, discovered during *post-mortem* investigations. Only eight of these children were already known to be victims of abuse [4]. Similarly, only 33% of children who were born in California between 1999 and 2006 and died from intentional injury during the first five years of life had been previously reported to Child Protection Services [5]. Consequently, children who died from child maltreatment can be victims of chronic child abuse while they were not diagnosed before their death. Systematic early detection of abused children could help prevent these deaths and lessen child maltreatment-related morbidity. However, as in usual screening programs, it is important to balance potential positive and negative effects and to determine the conditions for a screening program of child maltreatment to be effective. A first necessary condition is the availability of a test identifying correctly abused children before they have serious or irreversible consequences of maltreatment.

Diagnostic accuracy of ocular signs in abusive head trauma and clinical and neuroradiological features associated with abusive head trauma have been already synthesized [6-9]. In the reviewed studies, however, markers identified children when they had already serious consequences of child maltreatment. Sometimes the diagnosis had been done when the child was dead. Furthermore, the diagnostic accuracy of markers was not always estimated, the analysis being limited to estimating the association between a marker and maltreatment. Similarly, diagnostic accuracy of genital examination for identifying sexually abused prepubertal girls was reviewed [10], but tools only identified children who were victims of a severe form of sexual abuse (genital contact with penetration). Furthermore, the sensitivity for several potential markers, such as hymeneal transections, deep notches or perforations, was never reported.

Several authors have already considered screening in emergency departments [11-13]. A large study in the United Kingdom evaluated the accuracy of potential makers: child age, type of injuries, incidence of repeat attendance, and the accuracy of clinical screening assessments for detecting physical abuse in injured children attending Accident and Emergency departments [13]. They found no relevant comparative studies for incidence of repeat attendance, only one study which reported a direct comparison of type of injury in abused and non-abused children, and three studies for child age. However two of these three studies were limited to a subset of children admitted with severe injuries. Besides, assessments by the medical team were rarely based on standardized criteria, and therefore not reproducible and usable in practice [13]. The same team published another study about the same markers (age, repeated attendance, and type of injury) to identify children victims of physical abuse or neglect among injured children attending Emergency departments [14]. They found no evidence that any of the markers were sufficiently accurate. Thus these two large studies only reviewed the accuracy of tests for two types of child abuse among children who attended Emergency departments and already had injuries. A last study had initially the aim of evaluating the accuracy of tools identifying early abused children, but only reported an accuracy assessment of tools identifying high-risk parents before occurrence of child maltreatment [15].

The aim of our study was to review the evidence on the accuracy of instruments for identifying abused children during any stage of child maltreatment evolution before their death, and to assess if any might be adapted to screening, that is if accurate screening instruments were available. We define as instruments any reproducible assessment used in any types of setting.

## Methods

### Search strategy

#### Information sources and search terms

Electronic searches were carried using PUBMED database from 1966 to April 2012, PsycINFO database from 1970 to April 2012, SCOPUS database from 1978 to April 2012, PASCAL and FRANCIS databases from 1961 to April 2012, to identify articles published in French or English. Search terms used were *child abuse, child maltreatment, battered child syndrome, child neglect, Munchausen syndrome, shaken baby syndrome, child sexual abuse*, combined with *sensitivity*, *specificity*, *diagnostic accuracy*, *likelihood ratio*, *predictive value*, *false positive*, *false negative, validity, test validation*, and *diagnosis*, *measurement*, *psychodiagnosis*, *medical diagnosis*, *screening*, *diagnosis imaging, physical examination, diagnostic procedure, scoring system, diagnostic, scoring system, score, assessment* (Table 1).

**Table 1:** Search terms used to identify potentially eligible articles

**Database**	**Search terms**
PUBMED	(“child abuse” [Mesh] or “child maltreatment”)
	AND
	(“sensitivity and specificity” [Mesh] OR “sensitivity” OR “specificity” OR “diagnostic accuracy” OR “likelihood ratio” OR “predictive value” OR “false positive” OR “false negative”)
PsycINFO	(“battered child syndrome” OR “child abuse”)
	AND
	(“diagnosis” OR “measurement” OR “psychodiagnosis” OR “medical diagnosis” OR “screening”)
SCOPUS	(“child abuse” OR “child maltreatment” OR “child neglect” OR “battered child syndrome” OR “munchausen syndrome” OR “shaken baby syndrome”)
	AND
	(“diagnosis” OR “measurement” OR “screening” OR “diagnostic imaging” OR “physical examination” OR “diagnostic procedure” OR “scoring system”)
	AND
	(“predictive value” OR “diagnostic accuracy” OR “likelihood ratio” OR “sensitivity” OR “specificity”)
FRANCIS/PASCAL	(“child abuse” OR “child maltreatment” OR “child neglect” OR “child sexual abuse” OR “battered child syndrome” OR “munchausen syndrome” OR “shaken baby syndrome”)
	AND
	(“diagnosis” OR “measurement” OR “screening” OR “physical examination” OR “diagnostic” OR “scoring system” OR “score” OR “assessment”)
	AND
	(“test validation” OR “validity” OR “sensitivity” OR “specificity” OR “predictive value” OR “diagnostic accuracy” OR “likelihood ratio”)

#### Eligibility criteria

To be included in this analysis, articles had to 1) state as an objective to estimate at least one accuracy parameter (sensitivity, specificity, predictive value or likelihood ratio) of a test identifying abused children (persons under age 18); 2) include a reference standard to determine whether a child had actually been abused; and 3) describe the assessed test, e. g. when the authors presented the information and method to carry the assessment, and not only the result of this assessment. As there is no gold standard for detecting child maltreatment, we defined acceptable reference standards as: expert assessments, such as child’s court disposition; substantiation by the child protection services or other social services; diagnosis by a medical, social or judicial team using one or several information sources (caregivers or child interview, child symptoms, child physical examination, and other medical record review). The assessment made only by the caregiver was not accepted because 80% or more of maltreatment, other than sexual abuse, has been estimated to be perpetrated by parents or parental guardians [2]. Thus, the caregiver likely would not want to reveal that his child is maltreated. Comparative studies of any design examining the results of tools identifying abused children in two population groups (abused children and not abused children) were accepted (case control, cohort, and cross-sectional studies). Descriptive studies with only one group of abused or not abused children, of which the aim was to estimate one accuracy parameter, were also accepted. To avoid missing any potentially relevant tool, no particular setting nor category of patients were used as inclusion or exclusion criteria.

We did not consider tests to identify abusive caregivers, abused children after their death or children victims of intimate-partner violence. Articles were also excluded when they did not provide original data. Tests that identified abused children after their death were excluded as they are by definition not relevant for early detection. Intimate-partner violence, regarded as a separate form of child maltreatment by several authors, was excluded because the main victim is not the child [2].

#### Study selection

Eligibility of studies was checked by a junior epidemiologist and pediatrician (MB), from April, 2012 to May, 2012, and the resulting selection checked by a senior medical epidemiologist (LRS). Articles were first screened by titles. They were excluded when the title showed that the article did not address accuracy of tools identifying abused children. If the title did not clearly indicate the article’s subject, the summary was read. Abstracts were retained for full review when they met the inclusion criteria or when more information was required from the full text to ascertain eligibility.

#### Data collection process, data items and analysis

The first assessment of selected papers was done by MB, and results were discussed in regular meetings by both epidemiologists MB and LRS. To reduce the likelihood that potentially relevant articles were missed, reference lists from relevant articles were checked. From each included study, we abstracted information about study design, population characteristics, number of participants, screening instrument or procedure, abuse or neglect outcome, and estimates of diagnostic accuracy. Results were not mathematically pooled due to varying methods and types of child abuse identified.

### Quality assessment

The selected studies were assessed by MB and reviewed by LRS, using the QUADAS-1 criteria to assess quality of studies of diagnostic accuracy [16]. The standardized checklist included 15 criteria, grouped according to the domains defined by QUADAS-2 [17].

Two criteria related to patient selection:

1) patients were representative of a spectrum of population including all stages of maltreatment before the death of the child;

2) selection criteria were well described.

Three criteria related to the index test:

3) the index test was described in sufficient details to permit replication;

4) when the index test was a score, the cutoff was determined before results were available;

5) the index test was interpreted without knowledge of the results of the reference standard.

Three criteria related to the reference standard:

6) the reference standard correctly classified patients;

7) the reference standard was described in sufficient details to permit replication;

8) the reference standard was interpreted without knowledge of the results of the index test.

One criterion related to both the index test and reference standard:

9) the reference standard and the index test were independent.

Five criteria related to flow and timing:

10) the whole population or a random selection received the reference standard;

11) the study population received the same reference standard;

12) the time period between the reference standard and the index test was short enough so the situation of the child did not change;

13) uninterpretable test results were reported;

14) uninterpretable test results were well-balanced between the reference standard and the index test.

One criterion related to applicability:

15) same clinical data available when test results were interpreted as would be available when the test is used in practice.

Quality of studies was summarized by counting the number of criteria that were respected. Results of the final selection and analysis where reviewed by another senior medical epidemiologist (VL) and a senior pediatrician (PP).

### Assessment of tools adaptation to screening

Tools were considered adapted to screening, according to the WHO criteria on the adequacy of tests used in screening programs [18], if they fulfilled the following criteria: 1) identify abused children before they have serious consequences of child maltreatment; 2) identify abused children with a high sensitivity; 3) identify abused children with a high enough specificity to avoid stigmatization of caretakers who were not abusers.

## Results

### Study selection

**Figure 1:**
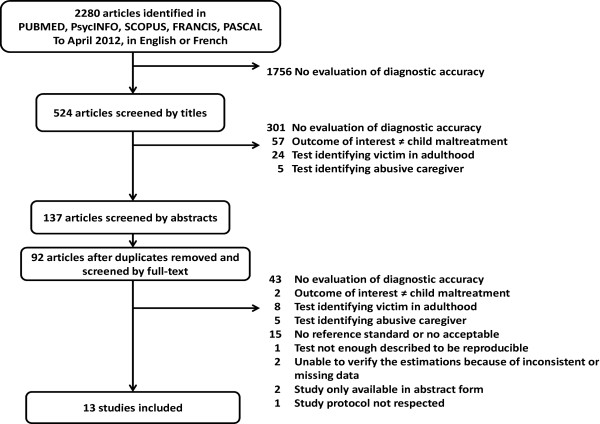
Diagram illustrating the study selection process, April 2012.

Of 2 280 references identified in the databases, 524 were selected from their title, of which 137 abstracts were read; after exclusion of duplicates, 92 full articles were assessed (Figure 1). Studies excluded for lack of reference standard were case–control studies with control groups recruited in the general population without verifying if children were abused or not. Studies were excluded when the reference standard was only the opinion of caregivers who had been asked whether their children were abused or not. One study was excluded because the method of the index text, an assessment by primary care clinicians, was not described [19]. Finally, one study was excluded because an unknown number of children less than fifteen years old examined in a medical center, who should have been tested during the study period, had not received the index test but were not registered [20]. This limit was noticed because several abused children identified by the reference standard and who had inclusion criteria, had not received the index test by the medical team and were not reported. Thirteen articles met the inclusion criteria. The outcome of interest was sexual abuse in four studies [21-24], physical abuse in seven [25-31], psychological abuse in one [32], and several forms of child maltreatment (physical abuse, psychological abuse, sexual abuse, and physical neglect) in one [33]. Eight studies were prospective [21-26,32,33], and five retrospective assessment of the diagnostic accuracy [27-31].

### Quality of studies

The maximum number of quality criteria met was eight of fourteen, and five studies met four or less criteria (Table 2). The accuracy of the reference standard was never determined because no gold standard to identify abused children is available. We could not judge patients representativeness, by lack of sufficient information about methods of patient recruitment [21,24,26,28,30-33], or refusal by many families, for undocumented reasons [22,23]. In three studies, details on the imaging technique or assessment of impact trauma were not sufficiently described to replicate the index test [25,27,28]. The reference standard was different in the three case–control studies [21,22,31]. In one study, the result of the index test was used to establish the final diagnosis [23]. The time period between the two tests was rarely available; in one study, it was on average 36.4 weeks, so that the situation about child abuse could have changed [33]. We could not judge if the circumstances of test evaluation were the same than in routine practice, by lack of information about the kind of practice considered [22,25-29,31,33].

**Table 2:** Quality of studies of the diagnostic accuracy of tests identifying child neglect or abuse

**Criteria of quality**	**Studies**												
	**Berenson et al, 2002 **[22]	**Bernstein et al, 1997 **[33]	**Chang et al, 2005 **[29]	**Cheung et al, 2004 **[23]	**Drach et al, 2001 **[24]	**Fernando-pulle et al, 2003 **[32]	**Hettler et al, 2003 **[27]	**Pierce et al, 2010 **[31]	**Valvano et al, 2009 **[30]	**Vinchon et al, 2010 **[25]	**Vinchon et al, 2005 **[26]	**Wells et al, 2002 **[28]	**Wells et al, 1997 **[21]
**1. Representative spectrum of patients**	Unclear	Unclear	Yes	Unclear	Unclear	Unclear	Yes	Unclear	Unclear	No	Unclear	Unclear	Unclear
**2. Description of selection criteria**	Yes	No	Yes	No	No	No	Yes	No	No	Yes	No	No	No
**3. Replication of the index test**	Yes	Yes	Unclear	Yes	Yes	Yes	No	Yes	Yes	No	Unclear	No	Yes
**4. Cutoff determined before results were available**	Yes	No	No	NA*	Yes	No	NA*	No	NA*	NA*	NA*	No	No
**5. Interpretation without knowledge of the results of reference standard**	Unclear	Yes	Unclear	Unclear	Unclear	Yes	Unclear	No	Unclear	Unclear	Unclear	Yes	Unclear
**6. Classification by reference standard**	Unclear	Unclear	Unclear	Unclear	Unclear	Unclear	Unclear	Unclear	Unclear	Unclear	Unclear	Unclear	Unclear
**7. Replication of the reference standard**	No	No	No	Yes	No	No	No	No	No	No	No	No	No
**8. Interpretation without knowledge of the results of index test**	Unclear	Yes	Unclear	Unclear	Yes	Yes	Unclear	Yes	Yes	Unclear	Unclear	Yes	Unclear
**9. Independence of reference and index tests**	Yes	Unclear	Unclear	No	Yes	Yes	Yes	Unclear	Yes	Unclear	Unclear	Unclear	Unclear
**10. Systematic reference standard**	Yes	Yes	Yes	Yes	Yes	Yes	Yes	Yes	Yes	Yes	Yes	Yes	Yes
**11. Same reference standard**	No	Yes	Yes	Yes	Yes	Yes	Yes	No	Yes	Yes	Yes	Yes	No
**12. Short enough time period between reference and index tests**	Yes	No	Yes	Unclear	Unclear	Unclear	Yes	Unclear	Unclear	Unclear	Unclear	Unclear	Unclear
**13. Uninterpretable results reported**	Yes	No	No	No	No	No	Unclear	No	No	No	No	No	No
**14. Uninterpretable results balanced**	Yes	Unclear	Unclear	Unclear	Unclear	Unclear	Unclear	Unclear	Unclear	Unclear	Unclear	Unclear	Unclear
**15. Same clinical data available as in routine**	Unclear	Unclear	Unclear	Yes	Yes	Yes	Unclear	Unclear	Unclear	Unclear	Unclear	Unclear	No

*NA Not Applicable.

### Diagnostic accuracy

#### Identification of physical abuse

Four studies were about children with inflicted head injury (Table 3) [25-28]. One test identified abused children among those admitted to a tertiary care pediatric hospital for acute traumatic intracranial injury, when caregivers reported no history of trauma or a history of low-impact trauma, i.e. with a fall from ≤ 3 feet or with other low-impact non-fall mechanisms [27]. The other tests identified abused children by using findings of physical examination or Computer Tomographic among children hospitalized in Pediatric Intensive Care Units [25,26], Neurosurgical [25,26] or Emergency departments [25,26] or a regional pediatric medical center [28] for head trauma. A prediction rule combining four variables (hygroma; convexity subdural hematoma without hygroma; no fracture; and interhemispheric subdural hematoma in Computer Tomographic images at clinical presentation) could identify 84% of abused children [28].

**Table 3:** Description of selected studies estimating diagnostic accuracy of tests identifying physical abused children

**Source**	**Inclusioncriteria**	**Form of child abuse**	**Indextest**	**Sample size**	**Referencestandard**	**Sensitivity** **% (95% CI)**	**Specificity** **% (95% CI)**
**Vinchon et al, 2010 **[25]	Children <2 y referred alive to Emergency, PICU* or ND† for HT‡ with cerebral scan	Inflicted head injury	Severe RH§	84	Assessment by forensic neurosurgeon, pediatrician, psychologist, social worker	57	97
			Brain ischemia			27	97
			SDH‖			27	97
			No scalp swelling			98	77
**Vinchon et al, 2005 **[26]	Children <2 y referred alive to Emergency, PICU* or ND† for HT‡ with cerebral scan	Inflicted head injury	RH § Grade 1, 2 or 3	207	Assessment by forensic neurosurgeon, pediatrician, psychologist, ophthalmologist, social worker	75(62-86)	93(85-78)
			RH § Grade 2 or 3			66(52-78)	100(95-100)
**Hettler et al, 2003 **[27]	Children < 3 y hospitalized for HT‡ with intracranial hemorrhage	Inflicted head injury	No history of trauma or low-impact trauma	163	Assessment by medical team integrating witnessed or confessed abuse, predefined specific findings during physical child examination	69(55-82)	97(83-100)
**Wells et al, 2002 **[28]	Children <3 y hospitalized for HT‡ with intracranial hemorrhage	Inflicted head injury	Score integrating CT¶ imaging patterns	257	Assessment by medical team, integrating history, age and sex of child, results of official investigation, medical records excluding CT¶	84(78-90)	83(74-90)
**Pierce et al, 2010 [**[31]	Newborn to 4 y hospitalized in PICU* for trauma	Physical abuse	Decision tool integrating bruise region, age of child, trauma history	95	Assessment by medical, juridical team, and CPS**	97(84-100)	84(69-94)
**Valvano et al, 2009 **[30]	Children <18 y referred to specialized team with fracture, excluded head	Physical abuse	Bruise in the same body sites†† than fracture	150	Expert assessment integrating history, type of injuries and familial characteristics	26(17-36)	75(62-86)
**Chang et al, 2005 **[29]	children ≤ 14 y with at least one trauma diagnostic with ICD-9‡‡	Physical abuse	SIPCA§§, score integrating age of child, physical examination and results of imaging	58 558	E codes and certain ICD-9 codes‡‡	87(84-90)	81(81-81)

*PICU Pediatric Intensive Care Unit.

† *ND* Neurosurgical Department.

‡ *HT* Head Trauma.

§ *RH* Retinal Hemorrhage.

§ *SDH* Subdural Hematoma.

¶ *CT* Computed Tomographic.

**CPS Child Protection Service.

†† Seven body sites: four extremities, torso, pelvis and head/neck.

‡‡ *ICD* International Classification of Diseases, Ninth Revision.

§§ *SIPCA* Screening Index for Physical Child Abuse.

Three studies estimated accuracy of tests identifying physical abuse and were not limited to intentional head trauma [29-31]. A decision tool based on three questions (age of child; localization of bruise during the initial 72 hours of patient’s admission; and confirmation of accident in public setting) identified abused children among children aged 0 to 4 y admitted to a Pediatric Intensive-Care Unit, with a sensitivity of 97% (95% CI: 84-100) [31]. In another study, presence of bruises in the same body site than a fracture identified 26% of abused children among children with acute fractures referred for possible child abuse to a specialized team [30]. Finally, a score was developed to identify physical abused children 14 years old or younger, with at least one diagnosis of injury as defined by the International Classification of Disease (ICD-9), 9^the^ revision (codes 800 to 959), in 1961 hospitals in 17 states of the United States. The 26-point score based on presence of fracture of base or vault of skull (1 point), eye contusion (3 points), rib fracture (3 points), intracranial bleeding (4 points), multiple burns (3 points), and age of the child (3 points for age group 1-3 y, 12 points for age group 0-1 y) identified 87% of physical abused child when the score was ≥ 3 [29].

#### Identification of sexual abuse

The sensitivity of tests using the results of children anal and genital examination were estimated at best at 56% (95% CI: 33-77), and the specificity at 98% (95% CI: 91-100) [22,23] (Table 4). The frequency of a variety of sexual behaviors of the child over the previous six months prior to assessment was not associated with sexual abuse [24]. A list of 12 symptoms expressed by the child, such as difficulty getting to sleep, change to poor school performance, or unusually interest about sex matters, identified sexual abused children when caretakers reported at least three symptoms, with a sensitivity of 91% and a specificity of 88% [21]. The setting in which the studies took place were consultations with specialized team in child abuse, or when a control group was chosen, consultations at pediatric clinics for well-child examination or others complaints.

**Table 4:** Description of selected studies estimating diagnostic accuracy of test identifying abused children, excluding physical abuse

**Source**	**Inclusion Criteria**	**Form of child abuse**	**Sample size**	**Index Test**	**Reference Standard**	**Sensitivity** **% (95% CI)**	**Specificity** **% (95% CI)**
**Cheung et al, 2004 **[23]	Children <18 y, referred to specialized team*	Sexual abuse	77	Classification of anal and genital examination findings	Assessment by medical team integrating medical history, children behavior, laboratory results, anogenital findings	56 (33-77)	98 (91-100)
**Berenson et al, 2002 **[22]	Girls 3-8 y referred to specialized team* or consulting at the pediatric clinics	Sexual abuse with penetration	386	Horizontal diameter of the hymen > or ≤ 6.5 mm in knee-chest position	Assessment by nurse, psychologist or social worker integrating children interview, CSBI† and assessment by CPS‡. Assessment by nurse integrating D/P vulvar Penetration Rating Scale§	29 (22-36)	86 (81-91)
**Drach et al, 2001 **[24]	Children 2-12 y referred to SCAP team‖	Sexual abuse	209	CSBI† parental interview about child sexual behavior	Expert assessment integrating child interview, history and physical examination	50 (37-63)	50 (42-58)
**Wells et al, 1997 **[21]	Boy < 18 y referred to CPS or consulting for well-child examination	Sexual abuse	74	SASA¶, parental interview integrating 12 child symptoms	Assessment by CPS or by a series of screening techniques	91 (71-99)	88 (77-96)
**Fernan-dopulle et al, 2003 **[32]	Children	Emotional abuse	98	Self-report questionnaire directed to children	Psychiatrist’s assessment during child interview	77 (56-91)	51 (39-63)
	13-15 y in school						
**Bernstein et al, 1997 **[33]	Children	Physical abuse	190	CTQ**, self-report questionnaire directed to children	Assessment by therapists integrating structured child interview, follow-up information and assessment of CPS†	82 (70-90)	73 (63-81)
	12-17 y hospitalized in psychiatry						
		Emotional abuse				79 (66-88)	72 (62-80)
		Sexual abuse				86 (71-94)	76 (67-83)
		Physical neglect				78 (62-89)	61 (53-70)

*Team evaluating children during reporting to Child Protection Services.

† *CSBI* Child Sexual Behavior Inventory.

‡ *CPS* Child Protection Services.

§ Score evaluation the probability of sexual penetration.

‖ Spurwink Child Abuse Program for identifying abused children in Oregon.

¶ *SASA* Signs Associated with Sexual Abuse.

**CTQ Childhood Trauma Questionnaire.

#### Identification of psychological abuse

In a self-administered questionnaire, children were expected to indicate how often they experienced a given parental/caregiver behavior (Table 4). The scale was administered to children aged 13-15 years without specific complaints attending a school within the city of Colombo. At a cutoff of 95 and greater, 20 of 26 abused children were identified [32].

#### Identification of several forms of child maltreatment

The Childhood Trauma Questionnaire is a 70-item screening inventory that assesses self-reported experiences of abuse and neglect in childhood and adolescence (Table 4). Accuracy was estimated for each form of child maltreatment in an adolescent psychiatric population. Physical neglect was defined as the failure of caretakers to provide for a child’s basic physical needs like food or clothing. The estimated sensitivity and specificity were the best for sexual abuse. The sensitivity were estimated at 86% (95% CI: 71-94), and the specificity at 76% (95% CI: 67-83) [33].

### Adaptation to screening

Identified tools were not adapted to screening because of low sensitivity and late identification of abused children when they have already serious consequences of maltreatment.

## Discussion

Assessment of the accuracy of instruments is difficult, because there is no gold standard for identifying abused children. To optimize the reference standard, opinion of experts or medical, social or judicial teams are usually used [21,24-28,30-33], but the accuracy of these assessments is not known. Furthermore, the information used for this assessment was rarely specified so that it was difficult to verify the independence between the index test and the reference standard. The incorporation of index test results in the reference standard would overestimate accuracy of the test [21,25,26,28,29,31,33]. Chang et al used the International Classification of Diseases (ICD), 9^th^ Revision, and E-codes (External cause), used to categorize intent and mechanism of an injury, for reference standard [29]. In a recent study in the Yale-New Haven Children’s hospital from 2007 to 2010, the specificity of coding injuries as physical abuse was 100% (95% CI: 96-100). But the sensitivity was low: among the 43 cases determined to be abused by the Child Abuse Pediatrician, four were miscoded as accidents, two as injuries of undetermined cause, and four did not receive any injury code [34]. In 1991-1992 in California, the sensitivity of hospital E-coded data in identifying child victims of intentional injuries had been estimated at 75% (95% CI: 64-84) [35]. This classification underestimates the number of abused children, therefore does not seem to be a good reference test. Cases of child physical abuse are considered as accidents and cases classified as physical abuse are not representative of all the cases of physical abuse, because some cases did not receive any injury code.

In this systematic review, the quality of selected studies was low, even when not considering the criterion related to the reference standard. Available information was often insufficient to make a judgment for many criteria. Some of the limitations, for instance the utilization of the index test to establish the final diagnostic, are particularly worrisome as they reflect an important misconception of what is good diagnostic research. This overall poor quality likely limits the validity of the selection of studies, as many could have been excluded on the basis of quality alone. Clearly, the quality of reporting of studies of diagnostic accuracy on child maltreatment needs to improve. Furthermore in five studies, the retrospective evaluation based on a review of records could have introduced bias [27-31]. And in the three case–control studies, the performance of index test could have been overestimated because of the increase of differences between both groups by excluding children for whom maltreatment is difficult to diagnose [21,22,31].

We were interested in tools identifying abused children as early as possible in the evolution of child maltreatment. Existing instruments reported to diagnose child maltreatment were not designed for screening. Many tools identify abused children when they have already clinical consequences of child maltreatment, such as head injury, fracture, or behavior problems [21,24-31]. The identification of abused children already at the clinical stage comes too late. The performance of tests was also not adapted to screening. Screening instruments require high sensitivity for missing very few abused children. In our synthesis, most sensitivity estimations were low [22-27,30,32,33]. Furthermore, the specificity of tests is also important because of the negative effects of a misidentification, in particular the psychological impact and the effect of a potential stigmatization on the child and his parents [36]. As usual, when the sensitivity of the test was high, the specificity was often low [25]. The sensitivity was greater than 90% and the specificity greater than 80% only for two tests [21,31]. However, one was a decision tool to identify physically abused children among those hospitalized in a Pediatric Intensive Care Unit, so that children had severe injuries [31]. The other test was based on twelve child symptoms to identify sexually-abused children [21]. These symptoms could be severe psychological consequences as depression: sudden emotional and behavior changes, changes to poor school performance, frequent stomachaches, difficulty getting to sleep or sleeping more than usual.

Child maltreatment is the “disease” of both the child and his caregiver. Obviously, an abusive caregiver is defined by his abusive behavior and child maltreatment begins by abusive behavior of caregiver. This abusive behavior is responsible for poor health and development of the child. Thus, identification of child maltreatment could consider the identification of both the abused child and his abusive caregiver. Two self-report questionnaires were directed to children who had to indicate if they had experienced given behaviors of parents or caregivers [32,33]. As only children old enough for reading could answer, these questionnaires cannot help reduce deaths in the most vulnerable groups. Indeed, fatal child maltreatment occurs most frequently when children are younger [2,37-39]. Over a half of the 600 victims of child maltreatment under five years reported to the National Violent Death Reporting System of the United States of America from 2003 to 2006 were under one-year-old [40].

The WHO definition of child maltreatment is problematic as it is defined by consequences of neglectful or abusive behaviors that, themselves, are not defined [1,3]. Similarly, the Article 19 of the United Nations convention on the rights of the child, stating “all forms of physical or mental violence, injury and abuse, neglect or negligent treatment, maltreatment or exploitation, including sexual abuse” does not define these behaviors. Moreover, proposed definitions based only on abusive behaviors can vary widely. For example, physical contact or penetration are applied before defining reported experiences as sexual abusive by some authors and not others [41-44]. Instruments designed to diagnose abusive caregivers such as the Child Abuse Potential Inventory [45], the International Society for the Prevention of Child Abuse and Neglect (IPSCAN) Child Abuse Screening Tool-Parent [46] measure these potential abusive behaviors of caregiver. Consequently, what they measure is not well known and defined. Furthermore they can identify only child maltreatment which is directly due to the questioned parent. These problems might explain why child maltreatment is usually recognized only when the child has consequences of abusive behaviors.

Due to the lack of knowledge of the evolution of child maltreatment, studying the accuracy of diagnostic instruments identifying abused children early remains challenging. Research is required to define what subclinical and clinical abusive behaviors are and when the child maltreatment begins. A multidisciplinary approach might be necessary to correctly identify child maltreatment because of its multiple targets, the child and the caregiver. Input from adult psychiatry is necessary to be able to assess the potential abusive behaviors of caregivers. One might reasonably hypothesize that tools based on simultaneous assessment of potential abusive behaviors and health and development of the child could allow earlier identification of abused child or abusive caregiver than tools based only on separate assessments of the child or caregiver. However, if a combined approach is likely to be more sensitive, it might also be less specific. Furthermore, because of the several types of child maltreatment and the varied consequences to children, several tests might be necessary to screen all types of child maltreatment. The final value of features used for screening will also depend on the prevalence of these features.

We reviewed studies only in French and English and only published studies in databases, and might have excluded interesting research. Also, one of our inclusion criteria was that the aim of the study was clearly to estimate the diagnostic accuracy of a test identifying abused children. This might have disqualified some studies in which some parameters of diagnostic accuracy could be estimated. Finally, we were interested in all forms of child maltreatment and all types of tools and we have not specified a particular such as emergency departments. Depending on the context, some tools could not be applied: for example a test requiring a specific laboratory result if the laboratory exam cannot be performed routinely. Besides, we reviewed the evidence on the accuracy of instruments for identifying abused children during any stage of child maltreatment evolution before their death. Thus both diagnostic and screening studies could be included in our review. We evaluated among the selected studies if accurate screening instruments were available. However the fact that screening test is sensitive and specific is not enough. The side effects, the reliability and the cost of the test should be also considered. Indeed before considering a screening program of child maltreatment, several other criteria need to be respected [18]. A screening program should also be acceptable to families and professionals. Negative effects for the family are consequences of false negatives (children identified wrongly as not abused) and of false positives (children identified wrongly as abused and parents identified wrongly as abusers). The stigmatization of families is an important ethical issue. Furthermore, confirming the relevance of screening of child maltreatment is not enough, as the modalities of the program should also be specified, including the site; the relevant target population group if screening is not mass screening, the child age at the time of screening, and the frequency if screening is repeated. At last, a screening program could become useless because of effective primary prevention program of child abuse. Several primary prevention programs, such as the Nurse Family Partnership [47] and the Early Start [48], have been proposed, but the evidence is currently insufficient to assess the balance between benefits and harms of primary care interventions [49].

## Conclusions

There is very scarce and low-quality evidence on the accuracy of instruments for identifying abused children. Child maltreatment is mostly identified when children have already serious consequences and the sensitivities and specificities of tools are inadequate. Before considering a screening program of child maltreatment, better knowledge on the beginning of child maltreatment and development of valid screening instruments at subclinical stages remain necessary.

## Abbreviations

E-code: External causes-code; ICD: International classification of diseases; WHO: World Health Organization.

## Competing interests

The authors declare that they have no competing interests.

## Authors’ contributions

MB conceptualized and designed the study, participated in the acquisition, analysis and interpretation of data, drafted the initial manuscript. VL participated in the analysis and interpretation of data, critically reviewed the manuscript. PP participated in the interpretation of data, critically reviewed the manuscript. LRS conceptualized and designed the study, participated in analysis and interpretation of data, drafted the initial manuscript. All authors read and approved the final manuscript.

## Pre-publication history

The pre-publication history for this paper can be accessed here:

http://www.biomedcentral.com/1471-2431/13/202/prepub

## References

[B1] World Health Organization Report of the consultation on child abuse prevention 1999 Geneva: World Health Organization, 1999 Document WHO/HSC/PVI/99.1

[B2] Gilbert R Widom CS Browne K Fergusson D Webb E Janson S Burden and consequences of child maltreatment in high-income countries Lancet 2009 13 9657 68 81 10.1016/S0140-6736(08)61706-7 19056114

[B3] Krug EG Dahlberg LL Mercy JA Zwi AB Lozano R World report on violence and health 2002 Geneva: World Health Organization

[B4] Tursz A Crost M Gerbouin-Rérolle P Cook JM Underascertainment of child abuse fatalities in France: retrospective analysis of judicial data to assess underreporting of infant homicides in mortality statistics Child Abuse Negl 2010 13 7 534 544 10.1016/j.chiabu.2009.12.005 20684844

[B5] Putnam-Hornstein E Report of maltreatment as a risk factor for injury death: a prospective birth cohort study Child Maltreat 2011 13 3 163 174 10.1177/1077559511411179 21680641

[B6] Bhardwaj G Chowdhury V Jacobs MB Moran KT Martin FJ Coroneo MT A systematic review of the diagnostic accuracy of ocular signs in pediatric abusive head trauma Ophthalmology 2010 13 5 983 992 e17 10.1016/j.ophtha.2009.09.040 20347153

[B7] Maguire S Pickerd N Farewell D Mann M Tempest V Kemp AM Which clinical features distinguish inflicted from non-inflicted brain injury? A systematic review Arch Dis Child 2009 13 11 860 867 10.1136/adc.2008.150110 19531526

[B8] Piteau SJ Ward MGK Barrowman NJ Plint AC Clinical and radiographic characteristics associated with abusive and nonabusive head trauma: a systematic review Pediatrics 2012 13 2 315 323 10.1542/peds.2011-1545 22778309

[B9] Kemp AM Jaspan T Griffiths J Stoodley N Mann MK Tempest V Neuroimaging: what neuroradiological features distinguish abusive from non-abusive head trauma? A systematic review Arch Dis Child 2011 13 12 1103 1112 10.1136/archdischild-2011-300630 21965812

[B10] Berkoff MC Zolotor AJ Makoroff KL Thackeray JD Shapiro RA Runyan DK Has this prepubertal girl been sexually abused? JAMA 2008 13 23 2779 2792 10.1001/jama.2008.827 19088355

[B11] Louwers ECFM Korfage IJ Affourtit MJ Scheewe DJH Van de Merwe MH Vooijs-Moulaert A-FSR Effects of systematic screening and detection of child abuse in emergency departments Pediatrics 2012 13 3 457 464 10.1542/peds.2011-3527 22926179

[B12] Louwers EC Korfage IJ Affourtit MJ De Koning HJ Moll HA Facilitators and barriers to screening for child abuse in the emergency department BMC Pediatr 2012 13 167 10.1186/1471-2431-12-167 23092228 PMC3502173

[B13] Woodman J Pitt M Wentz R Taylor B Hodes D Gilbert RE Performance of screening tests for child physical abuse in accident and emergency departments Health Technol Assess 2008 13 33 iii, xi-xiii 1 iii, xi-xiii 95 10.3310/hta12330 18992184

[B14] Woodman J Lecky F Hodes D Pitt M Taylor B Gilbert R Screening injured children for physical abuse or neglect in emergency departments: a systematic review Child Care Health Dev 2010 13 2 153 164 10.1111/j.1365-2214.2009.01025.x 20047596

[B15] Nygren P Nelson HD Klein J Screening children for family violence: a review of the evidence for the US preventive services task force Ann Fam Med 2004 13 2 161 169 10.1370/afm.113 15083858 PMC1466647

[B16] Whiting P Rutjes AWS Reitsma JB Bossuyt PMM Kleijnen J The development of QUADAS: a tool for the quality assessment of studies of diagnostic accuracy included in systematic reviews BMC Med Res Methodol 2003 13 25 10.1186/1471-2288-3-25 14606960 PMC305345

[B17] Whiting P Rutjes AWS Westwood ME Mallet S Deeks JJ Reitsma JB QUADAS-2: A revised tool for the quality assessment of diagnostic accuracy studies Ann Intern Med 2011 13 8 529 536 10.7326/0003-4819-155-8-201110180-00009 22007046

[B18] Wilson JMG Jungner G Principles and practice of screening for disease 1968 Geneva: World Health Organization

[B19] Sege R Flaherty E Jones R Price LL Harris D Slora E To report or not to report: examination of the initial primary care management of suspicious childhood injuries Acad Pediatr 2011 13 6 460 466 10.1016/j.acap.2011.08.005 21996468

[B20] Hammond J Perez-Stable A Ward CG Predictive value of historical and physical characteristics for the diagnosis of child abuse South Med J 1991 13 2 166 168 10.1097/00007611-199102000-00004 1990446

[B21] Wells R McCann J Adams J Voris J Dahl B A validational study of the structured interview of symptoms associated with sexual abuse (SASA) using three samples of sexually abused, allegedly abused, and nonabused boys Child Abuse Negl 1997 13 12 1159 1167 10.1016/S0145-2134(97)00091-4 9429768

[B22] Berenson AB Chacko MR Wiemann CM Mishaw CO Friedrich WN Grady JJ Use of hymenal measurements in the diagnosis of previous penetration Pediatrics 2002 13 2 228 235 10.1542/peds.109.2.228 11826200

[B23] Cheung PCH Ko CH Lee HYM Ho LMC To WWK Ip PLS Correlation of colposcopic anogenital findings and overall assessment of child sexual abuse: prospective study Hong Kong Med J 2004 13 6 378 383 15591595

[B24] Drach KM Wientzen J Ricci LR The diagnostic utility of sexual behavior problems in diagnosing sexual abuse in a forensic child abuse evaluation clinic Child Abuse Negl 2001 13 4 489 503 10.1016/S0145-2134(01)00222-8 11370722

[B25] Vinchon M De Foort-Dhellemmes S Desurmont M Delestret I Confessed abuse versus witnessed accidents in infants: comparison of clinical, radiological, and ophthalmological data in corroborated cases Childs Nerv Syst 2010 13 5 637 645 10.1007/s00381-009-1048-7 19946688

[B26] Vinchon M Defoort-Dhellemmes S Desurmont M Dhellemmes P Accidental and nonaccidental head injuries in infants: a prospective study J Neurosurg 2005 13 4 Suppl 380 384 15926388 10.3171/ped.2005.102.4.0380

[B27] Hettler J Greenes DS Can the initial history predict whether a child with a head injury has been abused? Pediatrics 2003 13 3 602 607 10.1542/peds.111.3.602 12612243

[B28] Wells RG Vetter C Laud P Intracranial hemorrhage in children younger than 3 years: prediction of intent Arch Pediatr Adolesc Med 2002 13 3 252 257 10.1001/archpedi.156.3.252 11876669

[B29] Chang DC Knight VM Ziegfeld S Haider A Paidas C The multi-institutional validation of the new screening index for physical child abuse J Pediatr Surg 2005 13 1 114 119 10.1016/j.jpedsurg.2004.09.019 15868569

[B30] Valvano TJ Binns HJ Flaherty EG Leonhardt DE Does bruising help determine which fractures are caused by abuse? Child Maltreat 2009 13 4 376 381 10.1177/1077559508326356 19001359

[B31] Pierce MC Kaczor K Aldridge S O’Flynn J Lorenz DJ Bruising characteristics discriminating physical child abuse from accidental trauma Pediatrics 2010 13 1 67 74 10.1542/peds.2008-3632 19969620

[B32] Fernandopulle S Fernando D Development and initial validation of a scale to measure emotional abuse among school children aged 13-15 years in Sri Lanka Child Abuse Negl 2003 13 10 1087 1099 10.1016/j.chiabu.2003.09.006 14602093

[B33] Bernstein DP Ahluvalia T Pogge D Handelsman L Validity of the childhood trauma questionnaire in an adolescent psychiatric population J Am Acad Child Adolesc Psychiatry 1997 13 3 340 348 10.1097/00004583-199703000-00012 9055514

[B34] Hooft A Ronda J Schaeffer P Asnes AG Leventhal JM Identification of physical abuse cases in hospitalized children: accuracy of international classification of diseases codes J Pediatr 2013 13 1 80 85 10.1016/j.jpeds.2012.06.037 22854329

[B35] Winn DG Agran PF Anderson CL Sensitivity of hospitals’ E-coded data in identifying causes of children’s violence-related injuries Public Health Rep 1995 13 3 277 281 7610215 PMC1382118

[B36] Teeuw AH Derkx BHF Koster WA Van Rijn RR Educational paper: detection of child abuse and neglect at the emergency room Eur J Pediatr 2012 13 6 877 885 10.1007/s00431-011-1551-1 21881926 PMC3357474

[B37] Sidebotham P Bailey S Belderson P Brandon M Fatal child maltreatment in England, 2005-2009 Child Abuse Negl 2011 13 4 299 306 10.1016/j.chiabu.2011.01.005 21481462

[B38] Herman-Giddens ME Brown G Verbiest S Carlson PJ Hooten EG Howell E Underascertainment of child abuse mortality in the United States JAMA 1999 13 5 463 467 10.1001/jama.282.5.463 10442662

[B39] Bennett MD Jr Hall J Frazier L Jr Patel N Barker L Shaw K Homicide of children aged 0-4 years, 2003-04: results from the national violent death reporting system Inj Prev 2006 13 Suppl 2 ii39 ii43 17170170 10.1136/ip.2006.012658 PMC2563475

[B40] Klevens J Leeb RT Child maltreatment fatalities in children under 5: findings from the national violence death reporting system Child Abuse Negl 2010 13 4 262 266 10.1016/j.chiabu.2009.07.005 20304491

[B41] Holmes WC Slap GB Sexual abuse of boys: definition, prevalence, correlates, sequelae, and management JAMA 1998 13 21 1855 1862 10.1001/jama.280.21.1855 9846781

[B42] Bassani DG Palazzo LS Béria JU Gigante LP Figueiredo ACL Aerts DRGC Child sexual abuse in southern Brazil and associated factors: a population-based study BMC Public Health 2009 13 133 10.1186/1471-2458-9-133 19432975 PMC2685133

[B43] Priebe G Svedin CG Prevalence, characteristics, and associations of sexual abuse with sociodemographics and consensual sex in a population-based sample of Swedish adolescents J Child Sex Abus 2009 13 1 19 39 10.1080/10538710802584635 19197613

[B44] Halpérin DS Bouvier P Jaffé PD Mounoud RL Pawlak CH Laederach J Prevalence of child sexual abuse among adolescents in Geneva: results of a cross sectional survey BMJ 1996 13 7042 1326 1329 10.1136/bmj.312.7042.1326 8646043 PMC2351043

[B45] Robertson KR Milner JS Convergent and discriminant validity of the child abuse potential inventory J Pers Assess 1985 13 1 86 88 10.1207/s15327752jpa4901_16 3989656

[B46] Runyan DK Dunne MP Zolotor AJ Madrid B Jain D Gerbaka B The development and piloting of the ISPCAN child abuse screening tool-parent version (ICAST-P) Child Abuse Negl 2009 13 11 826 832 10.1016/j.chiabu.2009.09.006 19854511

[B47] Olds DL Eckenrode J Henderson CR Kitzman H Powers J Cole R Long-term effects of home visitation on maternal life course and child abuse and neglect. Fifteen-year follow-up of a randomized trial JAMA 1997 13 8 637 643 10.1001/jama.1997.03550080047038 9272895

[B48] Fergusson DM Boden JM Horwood LJ Nine-year follow-up of a home-visitation program: a randomized trial Pediatrics 2013 13 2 297 303 10.1542/peds.2012-1612 23359575

[B49] Moyer VA on behalf of the U.S. Preventive Services Task Force Primary care interventions to prevent child maltreatment: U.S. preventive services task force recommendation statement Ann Intern Med 2013 13 4 289 295 10.7326/0003-4819-159-4-201308200-00667 23752681

